# Reduction in Asthma Morbidity in Children as a Result of Home Remediation Aimed at Moisture Sources

**DOI:** 10.1289/ehp.8742

**Published:** 2006-04-25

**Authors:** Carolyn M. Kercsmar, Dorr G. Dearborn, Mark Schluchter, Lintong Xue, H. Lester Kirchner, John Sobolewski, Stuart J. Greenberg, Stephen J. Vesper, Terry Allan

**Affiliations:** 1 Case Western Reserve University, Department of Pediatrics, Rainbow Babies and Children’s Hospital, Cleveland, Ohio, USA; 2 Cuyahoga County Board of Health, Parma, Ohio, USA; 3 Environmental Health Watch, Cleveland, Ohio, USA; 4 U.S. Environmental Protection Agency, National Environmental Research Laboratory, Cincinnati, Ohio, USA

**Keywords:** asthma, children, damp housing, home remediation, indoor mold

## Abstract

**Objective:**

Home dampness and the presence of mold and allergens have been associated with asthma morbidity. We examined changes in asthma morbidity in children as a result of home remediation aimed at moisture sources.

**Design:**

In this prospective, randomized controlled trial, symptomatic, asthmatic children (*n* = 62), 2–17 years of age, living in a home with indoor mold, received an asthma intervention including an action plan, education, and individualized problem solving. The remediation group also received household repairs, including reduction of water infiltration, removal of water-damaged building materials, and heating/ventilation/air-conditioning alterations. The control group received only home cleaning information. We measured children’s total and allergen-specific serum immuno-globulin E, peripheral blood eosinophil counts, and urinary cotinine. Environmental dust samples were analyzed for dust mite, cockroach, rodent urinary protein, endotoxin, and fungi. The follow-up period was 1 year.

**Results:**

Children in both groups showed improvement in asthma symptomatic days during the preremediation portion of the study. The remediation group had a significant decrease in symptom days (*p* = 0.003, as randomized; *p* = 0.004, intent to treat) after remodeling, whereas these parameters in the control group did not significantly change. In the postremediation period, the remediation group had a lower rate of exacerbations compared with control asthmatics (as treated: 1 of 29 vs. 11 of 33, respectively, *p* = 0. 003; intent to treat: 28.1% and 10.0%, respectively, *p* = 0.11).

**Conclusion:**

Construction remediation aimed at the root cause of moisture sources and combined with a medical/behavioral intervention significantly reduces symptom days and health care use for asthmatic children who live in homes with a documented mold problem.

Asthma is the single most common chronic disease of childhood, affecting > 3 million children in the United States. In addition, the burden of childhood asthma has increased over the past several decades despite the availability of excellent medications for controlling chronic symptoms and treating exacerbations. Moreover, asthma prevalence and morbidity are disproportionately high among inner-city children, most of whom are members of racial minorities. African-American children in the United States have a higher prevalence of asthma and greater morbidity as measured by acute care visits and hospitalizations compared with white children (Kattan et al. 1997; Mannino et al. 2002).

The role of the indoor environment in triggering and exacerbating asthma and other respiratory symptoms has been documented in several studies (Daisey et al. 2003; Engvall et al. 2001; Nafstad et al. 1998; Perry et al. 2003; Rosenstreich et al. 1997; Zock et al. 2002; Zureik et al. 2002). High exposures to dust mite, cockroach, and mold have all been implicated in producing respiratory illness, such as infections, cough, and wheeze. Home dampness and the presence of mold have also been associated with asthma, cough, and wheeze (Institute of Medicine 2004).

Dust mites, mold, and cockroach are indoor allergens and irritants related to home moisture content, and all are common in urban dwellings (Eggleston et al. 1999; Perry et al. 2003; Rosenstreich et al. 1997). High levels of indoor humidity promote the growth and survival of dust mites. Cockroaches can survive for long periods without food, as long as a water source, such as that from a leaky pipe, is available. Finally, growth of a number of indoor molds (*Aspergillus* species, *Cladosporium*, *Penicillium*, and *Stachybotrys*) may be promoted by high indoor humidity or water damage (Dales and Miller 1999). In addition to the allergen potential for mold, a number of fungal by-products, such as volatile organic compounds and β-1,2-glucans, may be respiratory irritants (Jaakkola et al. 2005; Rylander 2005). Inner-city children may be at increased risk for asthma morbidity due to exposure to such indoor allergens, because they often spend a large amount of time indoors and live in decaying housing stock that may be prone to water damage.

Previous indoor environmental asthma interventions focused on decreasing allergen/irritant exposure by either blocking exposure (use of dust covers) or reducing burden of specific allergen (cockroach abatement) (Evans et al. 1999; Gergen et al. 1999; Morgan et al. 2004; Shapiro et al. 1999; Woodcock et al. 2003). In this pilot program, we explored the effects of remediation of root causes of indoor home moisture and mold on asthma morbidity in inner-city children. Our hypothesis was that use of a home environmental intervention involving construction remediation of sources of moisture and mold damage would significantly decrease asthma symptoms and health care use beyond that achieved by use of a standard asthma intervention alone.

## Materials and Methods

Patients who were at risk for asthma morbidity as evidenced by previous, recent unscheduled health care use were targeted for the study. Patients were recruited during inpatient hospitalizations and after primary care visits and emergency department (ED) visits at Rainbow Babies and Children’s Hospital. Referrals were also accepted from a variety of community sources, including other health clinics, community health fairs, and general advertising. The study was conducted through the Center for Chronic Conditions of Childhood, a clinical research facility at Rainbow Babies and Children’s Hospital. The Institutional Review Board of University Hospitals of Cleveland approved the study protocol, and written informed consent was obtained from parents. Children were eligible if they were between 2 and 17 years of age at the time of recruitment, had physician-diagnosed asthma for at least 3 months before enrollment, were English speaking (child and caregivers), and had at least two ED visits or at least one hospitalization for asthma in the 12 months preceding enrollment. Families were also required to live in Cuyahoga County and agreed to remain in their current residence for the 12-month duration of the study.

Patients were excluded if there was a history of life-threatening asthma, including history of respiratory failure, intubation, hypoxic seizure or anaphylaxis, another respiratory illness such as cystic fibrosis, or any other chronic illnesses. Screening for the presence of household mold was performed by trained sanitarians and conducted during a home survey before enrolling the family in the program. Subjects who resided in households in which no visible mold was identified during the first environmental visit were also excluded.

Demographic and illness information was collected on all patients who were approached for recruitment into the study to determine eligibility, and application materials were completed at a first screening encounter [environmental visit 0 (EV0)]. Enrolled patients returned for the first study visit [clinical visit 0 (CV0)] and, after signing informed consent, had baseline study measures recorded (Table 1), including asthma symptoms, caregiver report of treatment plan, school asthma management, family responsibility for asthma, and caretaker brief symptom inventory. Spirometry was also obtained.

Using each subject’s reported asthma symptoms during CV0, a pediatric pulmo-nologist (C.M.K.) assigned the appropriate asthma severity category and formulated a written asthma treatment plan for both baseline and symptomatic periods, using criteria set forth in the National Asthma Education and Prevention Project guidelines (National Institutes of Health–National Heart, Blood, and Lung Institute 1997). The written treatment plan was provided to all patients during the initial study visit. A copy of this written treatment plan was forwarded to each enrolled subject’s primary care physician, who continued to provide asthma care throughout the study.

All patients received in-depth instruction on the use of their personalized medical treatment plan, as well as instructions for medication use, including use of spacer devices with metered-dose inhalers. During the next study visit, problem solving issues were discussed, based on data collected during baseline interviews. Barriers to adherence to the asthma management plan were explored and personalized, and specific interventions were provided for the patient and caregiver. A 24-hr telephone hotline, answered by trained nurses or physicians, was made available to all patients for questions regarding acute asthma management. At the next clinical visit (CV1), 1 month after baseline, families and children again were interviewed regarding asthma management and symptoms, using a standardized data collection instrument based on the Children’s Health Survey for Asthma (CHSA). The CHSA was designed, tested, and validated by the American Academy of Pediatrics to capture asthma symptoms, morbidity measures, and quality of life measures (Asmussen et al. 1999). Spirometry was again performed, and laboratory measures [complete blood cell counts, serum immunoglobulin E (IgE), radioaller-gosorbent test (RAST), urinary cotinine] were obtained. These first study visits and intervention measures were designed to provide optimized medical care, asthma knowledge, and management skills to all participants. Within 1 week of clinical evaluation (CV1), a home visit (EV1) was then performed by trained sanitarians for the purpose of determining the extent of household mold and moisture problems and to determine that remediation was feasible. A standardized visual assessment tool was used to score the extent of visible mold present in multiple areas of the home. Dust samples were obtained from the child’s bedroom to measure mold, dust mite, cockroach, mouse, and rat urine allergens and endotoxin. Dust was processed and assayed for allergens and endotoxin using standard methods by IBT Reference Laboratory (Lenexa, KS). A dust aliquot was analyzed by quantitative polymerase chain reaction for 33 fungal species using species-specific genomic probes (Vesper et al. 2004). Full details of the home survey procedures are described elsewhere (Environmental Health Watch 2005).

We calculated geometric means of spore-equivalent counts per square meter for indoor and outdoor molds species groups and for all 33 molds. The difference in the log of the geometric means between indoor and outdoor mold groups was also calculated. The 33 mold species were divided *a priori* into primarily indoor molds (*Aspergillus*- and *Penicillium*-predominant species) and outdoor molds (*Alternaria* and *Cladisporium*-predominant species). A full list of indoor and outdoor molds is available from the authors.

Randomization occurred immediately after EV1 if mold was identified, and subjects were stratified by age of the child, using a random permuted block scheme that prevented personnel from randomizing based on predictions of the next assignment. Once a child was determined to be eligible for randomization, study personnel accessed the program to obtain the next group assignment. Families randomized to the remediation group had a home remediation performed 4–5 months after study entry, after the extent of the repairs was assessed and a plan for construction devised (full details described elsewhere; Environmental Health Watch 2005). Briefly, interventions were directed at reducing water infiltration, removal of water-damaged building materials, alterations to heating/ventilation/air conditioning, lead hazard control, and environmental cleaning. General strategies included cleaning mold from hard surfaces, removing mold exposure pathways, stopping rainwater intrusion, exhausting water vapor from kitchens and bathrooms, and repairing plumbing leaks. Specific interventions included repair of faulty cold-air return to furnace, elimination of subslab heating duct systems, disconnecting and redirecting downspouts, and reducing moisture in crawlspaces and basements. After satisfactory home remediation, a date of clearance was issued, and the family and household entered the remainder of the follow-up phase of the study. A repeat home environmental survey for dust sampling occurred at approximately 6 and 12 months after randomization (EV2, EV3). However, because of unforeseen delays in completing home remediation, some visits were delayed > 6 months after due dates. Families randomized to the control group were given information on how to improve home indoor air quality, but were given no specific tangible resources, materials, or advice to do so (Environmental Health Watch 2005; U.S. Environmental Protection Agency 2002). At the end of the study, participants in the control group were given a vacuum cleaner and offered home remediation. Neither the study personnel nor the families were blinded as to group assignment once the intervention was started.

Telephone follow-up calls occurred at 2, 4, 8, and 10 months and follow-up visits at 6 and 12 months after randomization. During phone follow-up at 2, 4, 8, and 10 months, all subjects had interim asthma symptoms monitored using an abbreviated version of the CHSA. Caregivers were asked to recall frequency of symptoms (days and nights with wheezing, shortness of breath, cough, and difficulty sleeping during the previous 4 weeks). Caregivers were also asked to recall any hospitalizations, ED visits, unscheduled office visits, and missed school days during the previous 2 months. At the follow-up clinical visits (CV2 and CV3), the children also performed spirometry and certain laboratory tests. Testing included complete blood cell count with differential; serum lead concentration on children < 6 years of age; serum IgE; RAST for molds (*Aspergillus*, *Alternaria*, *Penicillium*, *Stachybotrys*), cockroach (Bla g 1), dust mite (Der p 1, Der f 1), mouse urine, and rat urine; and urinary cotinine.

Adverse events were noted for all entered subjects. Expected adverse events were asthma exacerbations requiring either unscheduled acute care visits (ED or urgent care) or hospitalizations. Any event requiring emergency or hospital care was recorded. Quarterly review of adverse events was monitored to assure that there was no discrepancy between the groups that may have required stopping the study. The number of asthma-related acute care visits (ED visits and hospitalizations) of all subjects were determined by self-report and confirmed by a review of hospital records.

### Statistical analyses

We compared categorical variables between groups using chi-square tests, using an exact test when sample sizes were small. We compared continuous variables between groups using *t*-tests or Wilcoxon rank sum tests. The primary outcome was symptomatic days, defined as maximum number of days when any asthma symptom (cough or wheeze) or asthma attacks occurred over the 4 weeks preceding the follow-up call or clinical visit. Secondary outcomes included CHSA subscale scores, health care use (ED visits and hospitalizations for asthma), inflammatory markers (serum and serum IgE), and home environmental markers (mold). Analyses were carried out in SAS/STAT software (versions 8.2 and 9.1; SAS Institute Inc. 2004a, b).

Because of unavoidable circumstances, three subjects originally randomized to the control group had home remediation performed and were included in the analysis as part of the remediation group; in addition, three subjects originally randomized to the intervention group did not have home remediation and were included in the analyses as control subjects. The groups are therefore referred to as “as-treated” to distinguish from as randomized. Data for the primary outcome and select secondary outcomes where differences occurred are given for both “as-treated” and “as-randomized” (intent-to-treat) analyses.

We used linear mixed-model analyses to compare symptom days and CHSA subscales. To better meet the assumptions of normality, we transformed symptom days by taking the natural logarithm of symptom days + 1, where the constant 1 was added to avoid taking the logarithm of zero. We used a compound symmetry covariance structure in fitting the model; we estimated SEs and tests using an estimated covariance matrix of parameter estimates that is robust to misspecification of the form of the covariance structure. Estimated means and confidence intervals (CIs) from this were transformed back to the original scale. The model compared as-treated groups across visits, including baseline CV0 asthma severity as a baseline covariate and also adjusting for season of the year when the visit was held as a time-varying categorical covariate with four levels [winter (December–February), spring (March–May), summer (June–August), fall (September–November)]. The model also included terms for visit, group, and group × visit interaction. Data from baseline and all follow-up visits were included in the model. A *p*-value of 0.05 was considered significant.

We calculated the mold score by adding mold scales across all rooms, which included the basement, kitchen, TV/living room, bathroom, child’s bedroom, attic, and other bedroom. The visible mold scales in each room have four categories (0, none; 1, < 4 ft^2^; 2, 4–32 ft^2^; 3, > 32 ft^2^). We analyzed changes in mold scores from EV1 to EV2 using a mixed model, adjusting for season of the year.

We compared allergen levels and mold indices determined from dust samples between remediation groups using the Wilcoxon rank-sum test at EV1, EV2, and EV3. Changes from EV1 to EV2 and EV3 were tested within groups and compared between groups using a mixed linear model adjusting for season of the year and type of surface (carpet vs. hard).

The initial recruitment goal was 150 subjects, with 75 in each group, which initial power calculations indicated would provide 80% power to detect a difference between groups in the primary outcome of mean symptom days of 2.3 days per 4-week period with a two-sided test with α = 0.05. However, in spite of aggressive recruitment measures, it was not possible to reach the required number of subjects.

## Results

### Study population

There were 366 referrals made for study recruitment; of these, 261 completed EV0 and 62 were randomized. Subjects who did not enter the program did so for a number of reasons: 23.8% (87) had no mold detected, 4.4% (16) were moving, 23.2% (85) did not complete an application, and 0.8% (2) landlords refused to participate. Of the 62 patients enrolled, 82% (26 of 29 in the as-treated group receiving remediation; 25 of 33 in the group without remediation) completed the study, defined as completing the CV3 (12-month) visit.

Demographic data are shown in Table 2. There were no significant differences between the groups with respect to age, race, sex, or asthma severity. The mean age of the children was 7 years, and most were African American. More than 75% of the children had mild to severe persistent asthma. One-third of the families had an annual income < $20,000, and > 75% of families in both groups resided in traditional, single-family housing. Most families reported relatively few smokers in the household, with 73% of the control group reporting no smokers and 66% of the remediation group reporting no smokers. Mean (± SD) urinary cotinine levels measured at baseline were relatively low in both groups (control, 18.6 ng/mL ± 30.1; remediation, 31.9 ng/mL ± 40.4; *p* = 0.36). Fewer than 20% of families reported an obvious problem with roach or rodent infestation, and fewer than half of the households kept a pet. Total serum IgE concentrations were similar at baseline between the control and remediation groups, but peripheral blood eosinophil counts were significantly higher in the remediation group. There was no significant difference in the IgE levels (log total IgE IU/mL) or total eosinophil counts between the first and last clinical visits in either the control or remediation groups (either as randomized or as remediated). Examination of RAST results at baseline showed that there was no difference in the number of patients with positive tests to any mold, roach, dust, rat, or mouse urine. There was a trend for more patients to have any positive RAST in the remediation group compared with control, but this did not reach statistical significance. There was no difference in the reported use of controller medications (inhaled steroids and montelukast) between the control and remediation groups at CV1, CV2, or CV3 (data not shown).

### Environmental measures

Allergen and endotoxin concentrations (per square meter) measured throughout the study are shown in Table 3. There were no significant differences in any allergen or endotoxin measures at baseline between the control and remediated groups. We examined the relationship between baseline endotoxin levels and the presence of pets or pests in the home. No correlation existed between the presence of a pet and endotoxin levels. However, there was a signifi-cant correlation between mouse allergen levels (Spearman *r* = 0.30, *p* = 03), but not rat (Spearman *r* = 0.23, *p* = 0.10), and endotoxin. The mean change in endotoxin concentration between baseline and the EV2 sample was sig-nificantly greater in the remediated compared with the control group; however, the difference was no longer significant at EV3 (Table 3). There were no other significant changes in mean allergen levels between the groups at any time points. There was a trend toward a greater reduction in mouse allergen levels in the remediated group compared with control at EV3 and a similar trend toward greater β-glucan reduction in the control group compared with remediation at EV3 (data not shown). Significant within-group reduction was seen at EV3 for mouse urine in the remediated group. Der p 1 levels were significantly increased at EV2 in the control group.

Total mold scores were determined from the visual inspection performed by the sanitarians during the home environmental visits. Baseline mold scores were not different between the control and remediation groups. Total visible mold scores were significantly lower in the remediation group compared with the control at both EV2 and EV3 (Table 4). Finally, the changes in mold scores from baseline to EV2 and EV3 were greater in the remediation group compared with control, and approached statistical significance, with the direction of the change showing greater reduction in the remediation group.

Change in mold indices (measured as ln geometric mean of indoor and outdoor molds) over the course of the intervention was signifi-cantly greater in the remediation group compared with the control group for indoor but not outdoor species (Table 5). Although the change in the ratio of indoor to outdoor mold and the total fungal load between groups over the intervention period was reduced in the remediation compared with the control group, the difference did not reach statistical significance.

### Asthma symptoms and health care use

**Figure 1:**
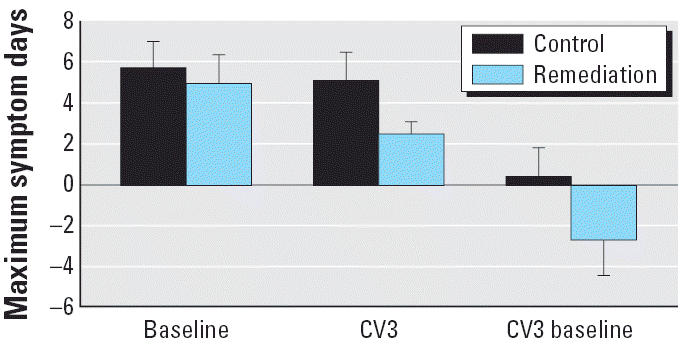
Unadjusted mean maximal symptom days ± 1 SE for control group and remediation group.

**Figure 2:**
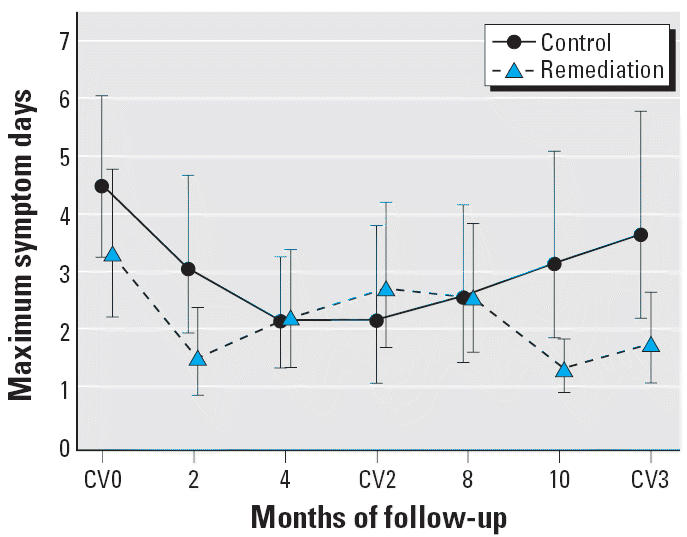
Mean maximal symptom days over study duration for control and remediation groups. Values are means and 95% CIs estimated from a mixed model, adjusting for baseline asthma severity and season of the year. In the remediation group, maximal symptom days decreased significantly from baseline at 10 months (*p* < 0.0001) and at CV3 (*p* = 0.053), whereas the changes from baseline in the control group at these time points were not statistically significant. Changes from baseline at 10 months and at CV3 did not differ significantly between control and remediated groups.

There was no difference in the maximum number of symptom days reported at baseline in the two groups. Although subjects in the remediation group reported fewer symptom days at the last follow-up visit compared with those in the control group, the differences did not reach statistical significance in univariate analyses (Figure 1). There was a greater reduction in symptom days in the remediation group compared with the control group when comparing baseline with CV3; but again, the difference was not statistically significant. However, in the mixed model analysis adjusted for baseline asthma severity and season, the remediation group showed a significant reduction in symptom days when comparing baseline with the 10-month follow-up (*p* = 0.0001), the last visit (*p* = 0.05), or the average of the 10-month and last visit values (*p* = 0.003) (Figure 2). In the as-randomized analysis, there were similar and statistically significant reductions from baseline to 10 months (*p* = 0.0002) and in the average of the 10-month and final values (*p* = 0.004). The reduction in symptom days from baseline to the final value (CV3) approached but did not reach statistical significance (*p* = 0.06). Changes in symptom days within the control group were not statistically significant.

Over the 12-month follow-up after randomization, 36.4% of controls versus 17.2% of subjects in the as-remediated group had one or more acute care visits (*p* = 0.15, Table 6); results for the as-randomized analysis were similar. Focusing on the period from 6 to 12 months postrandomization, which corresponded approximately to the postremediation period, 28.1% and 10.0% of subjects in the control and remediation groups (as randomized), respectively (*p* = 0.11), compared with 33.3% and 3.5% (*p* = 0.003) for the as-treated groups, had one or more acute care visits. Thus, although a similar trend existed in the as-randomized analysis, it was not significant at the 0.05 level. The 11 subjects in the control group made a total of 17 acute care visits, compared with two visits made by a single subject in the remediation group. There was minimal use of the hotline over the study duration; eight patients (four control, four remediation) made a total of 12 calls. Advice given was to follow action plan for eight calls, and to contact primary care physician for four calls. No patient was advised to go directly to the hospital by the hotline staff.

Pulmonary function data were available on a limited number of study subjects (*n* = 33) largely because of the young age of half the subjects and inability to perform acceptable spirometry. There was no difference in any spirometric measure at CV1 between the control and remediation groups. At the first postremediation visit (CV2), forced vital capacity, forced expiratory volume at 1 sec, and peak expiratory flow rate values were all higher in the remediation group than in the controls. At the end of the study (CV3), there was again no significant difference in any measure of pulmonary function between the groups (data not shown). However, the extremely small sample size limits the conclusions and increases potential biases related to these data.

**Figure 3:**
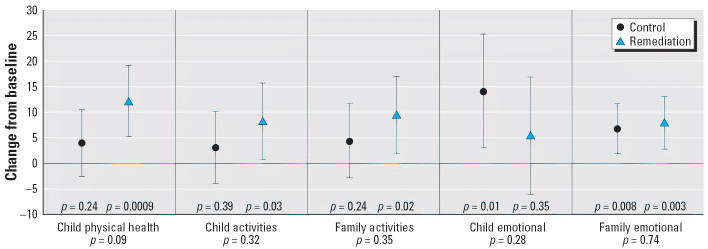
Changes from baseline to CV3 in CHSA subscales. Values are means and 95% CIs estimated from a mixed model, adjusting for baseline asthma severity and season of the year. *p*-Values above the *x*-axis, within the sections, are within-group comparisons; those below the section headings are between-group comparisons.

The CHSA measures functional morbidity in several domains, including asthma symptoms (child physical health), activity limitation of the child and family, and emotional well-being of both the child and the family. We saw no difference between the groups for any sub-scale measurement of the CHSA when comparing changes between baseline and CV3 (Figure 3). In the remediated group, there were significant within-group improvements in all domains, except for child emotional function. However, in the control group, there was significant improvement in only the child and family emotional domains.

### Cost

The mean (± SD) cost of remediation per household was $3,458 ± $2,795 (median, $3,182; range, $535–6,550).

## Discussion

We developed an intervention designed to examine the health effects of an environmental remediation aimed at reducing indoor mold and moisture for children with persistent asthma. Our sample was largely African American, and more than a third were indigent; these children represent a high-risk group for asthma morbidity. In addition, most were atopic, having at least one positive RAST result for an indoor allergen.

Because appropriate asthma management requires actions in several domains, such as medical care, family and patient education, management skills, adherence to treatment, and trigger and allergen avoidance, it was important to provide participants with adequate skills and tools in all the above areas to isolate the effects of the environmental intervention. By providing subjects in both the remediation and control groups with a multidisciplinary asthma intervention that we have used extensively in previous studies, our data on the effects of the environmental intervention on asthma morbidity have increased validity.

Previous studies have established the relationship of home dampness or mold to the presence of respiratory symptoms, such as bronchitis, cough, and other chest illnesses, but other studies have not (Daisey et al. 2003; Zureik et al. 2002). In a more recent study, exposure to household molds early in life was associated with the development of wheeze and persistent cough among children with a maternal history of asthma (Belanger et al. 2003). Some studies (Jaakola et al. 2005; Zock et al. 2002) relied on self-report of household dampness or the presence of mold, and this may have accounted for some of the variability in results. In the early phases of our study, we found that families were highly inaccurate in reporting the presence of mold, and we quickly adopted a strategy that permitted study randomization only after a direct home inspection confirmed the presence of mold. In addition, we used a visual assessment tool applied by a trained sanitarian to quantify the extent of mold present in the household.

Almost a third of subjects in each group had a positive RAST result for any mold. Moreover, 20% had a positive RAST for cockroach and > 25% for dust mite. The availability of water sources promotes the survival and reproduction of roaches, whereas high levels of indoor humidity are associated with dust mite and mold proliferation. Although outdoor molds can also be amplified with indoor damp conditions, the failure of the remediation to sig-nificantly reduce these molds may indicate that mold was tracked in from outdoors. The basement was most commonly the largest source of mold in the houses; therefore, the exclusion of basement air from the living space ventilation in 38% of the remediated homes likely contributed to the decrease in the amount of indoor molds in the children’s bedroom dust.

Our data suggest that in homes with a documented mold problem, a construction remediation aimed at the root cause of the moisture sources significantly reduces symptomatic days for the asthmatic children living in those homes compared with asthmatic children living in homes without a mold remediation. Symptom days slightly decreased in both groups in the interval before remediation, suggesting an effect of the global intervention provided to both groups and/or regression to the mean. However, in the interval after remediation and by 10 months after study entry, subjects in the remediation group showed a significant reduction in maximum symptom days compared with baseline, whereas the control group did not. This symptom reduction persisted through the last study visits. In addition, there was also a marked reduction in ED visits and hospitalizations for asthma in the remediation group compared with the control group. Although acute care visits for asthma are relatively infrequent events, they are costly, disruptive, and harmful for children and families. Pulmonary function was also improved by CV2 in the remediation group but not in the controls; although promising, these data are limited by the relatively small number of participants who completed the tests.

Home remediation for environmental allergens and irritants has met with mixed success in previous studies. The National Cooperative Inner City Asthma Study applied medical, psychosocial, and environmental interventions in an attempt to decrease asthma morbidity in urban-living, school-age asthmatic children. A significant decrease in symptomatic days and health care visits was documented; however, no significant reduction in home environmental allergens and irritants (dust, cockroach) occurred, and only half of the enrollees obtained asthma treatment plans (Evans et al. 1999; Gergen et al. 1999). More recently, the Inner City Asthma Study demonstrated that an intensive, customized home intervention that included provision of pillow and mattress dust covers, room HEPA filters, vacuum cleaners, and integrated pest management services resulted in a decrease in indoor dust and roach allergen levels and a significant reduction in days with wheeze over a 2-year follow-up (Crain et al. 2002; Morgan et al. 2004). No structural repairs were conducted. Prior cockroach abatement programs used professional exterminators or an integrated pest management program to reduce roach allergen levels. Although professional extermination coupled with clean-up instructions for the family was not successful in significantly reducing roach allergen levels, the integrated pest management strategy was beneficial (Eggleston et al. 1999; Rosenstreich et al. 1997). The latter did involve removing water sources, such as plumbing leaks.

Compared with previous studies, our intervention took a more global approach to addressing root cause of home water damage and moisture sources that contribute to mold, roach, and dust mite problems. In addition, any moldy materials were removed from the homes during the remediation process. These types of repairs are often outside the financial resources of many families and are often ignored by landlords. We demonstrated a significant reduction in mold scores in the remediated homes compared with the control homes (Tables 4 and 5) using interventions of rather modest expense. These data support the relationship between reduction in home mold and moisture and improvement in asthma symptoms.

Our study has several limitations. First, the sample size was far lower than that required by our original study design and power analysis. Failure to recruit adequate numbers of families occurred for a number of reasons, such as the frequency with which inner-city families relocate and unwillingness to participate by landlords and, to a lesser extent, homeowners. The application process required a number of steps to complete and documents to produce (e.g., proof of home ownership, tax bills) that may have been too complex or invasive for some families. Because our sample size was relatively small, these data should be regarded as exploratory, and larger trials will be needed to confirm our results. Nevertheless, we believe the data strongly indicate that mold and moisture reduction are feasible and, when combined with other asthma intervention measures, can further reduce asthma morbidity in children. Further data analyses on the relationship of spe-cific fungi to asthma symptoms are also being conducted and will be reported elsewhere. A detailed cost analysis of the program will also need to be completed to help better guide the cost-effectiveness of the program. Although our data suggest that the home remediation remains intact and effective for at least a year after completion, it will be important to continue to maintain homes free from excessive moisture sources to prevent return of mold problems. Finally, other factors not obviously measured in our study besides reduction of household mold could have contributed to the improvement in asthma symptoms seen in the remediation group. Families in this group may have taken other steps to improve the indoor environment or otherwise improve the asthma care provided to their children as a result of being in the remediation group. Although the participants in both groups received the same degree of asthma intervention in terms of education, treatment plan, and problem solving, the control group subjects may have sought additional outside help for asthma or home remediation.

In summary, we have demonstrated in this small study the feasibility of performing successful home remediation for mold and moisture and the resultant improvement in asthma morbidity associated with reduction in indoor mold. We believe these data are strong enough to warrant further studies on home mold remediation to improve the health of children with respiratory conditions. Future studies, particularly with inner-city populations, will need to use streamlined, simple recruitment and entry criteria to ensure that those in greatest need participate.

## Figures and Tables

**Table 1:** Study timeline and schedule of measures (days).

Visit	EV0 −30 to 1	CV0 11–14	PS 15–30	CV1 31–45	EV1 52	R/C 120–150	C2 150	C4 210	CV2, EV2 270	C8 330	C10 390	CV3, EV3 450
Recruitment	X											
Home mold screen	X											X
Consent		X							X			
Clinical testing		X		X								X
Spirometry		X		X					X			
Home environment									X			X
Sampling					X							
Problem solving			X									
Randomization					X							
Remediation						X						
Clearance						X						
Phone follow-up							X	X		X	X	

Abbreviations: C, call; PS, problem-solving visit; R/C, remediation/clearance.

**Table 2:** Demographic and immunologic data.

Characteristic	Control (*n* = 33)	Remediation (*n* = 29)
Age [years (mean ± SD)	6.5 ± 3.9	7.1 ± 3.8
Male sex [*n* (%)]	18 (54.5)	19 (65.6)
Ethnicity [*n* (%)]		
Black/other	23 (71.9)	24 (82.8)
White	9 (28.1)	5 (17.2)
Insurance [*n* (%)]		
Medicaid/self	17 (58.6)	13 (54.1)
Private	12 (41.4)	11 (45.8)
Severity [*n* (%)]		
Intermittent	8 (24.2)	5 (17.2)
Mild	16 (48.5)	14 (48.3)
Moderate	6 (18.2)	6 (20.7)
Severe	3 (9.1)	4 (13.8)
Housing type [*n* (%)]		
Traditional	28 (84.8)	23 (82.1)
Section 8/other	5 (15.2)	5 (17.9)
Number living in home (mean ± SD)	4.4 ± 1.7	4.9 ± 1.6
Roach problem in last year [*n* (%)]	6 (18.2)	4 (13.8)
Rodent problem in last year [*n* (%)]	6 (18.2)	4 (13.8)
Pet (any type) in home [*n* (%)]	14 (43.8)	10 (35.7)
CV1 blood eosinophil % [mean ± SD (*n*)]	3.8 ± 3.6 (28)	7.2 ± 5.6 (23)*
Serum IgE [log_10_(total IgE)] baseline [mean ± SD (*n*)]	2.13 ± 0.77 (26)	2.09 ± 0.78 (22)
Baseline RAST by study group (%)		
Any RAST	13/32 (40.6)	18/27 (66.7)
Any mold	10/32 (31.3)	9/27 (33.3)
Cockroach (German)	6/27 (22.2)	4/22 (18.2)
*Dermatophagoides pteronyssinus* mite	8/31 (25.8)	10/27 (37.0)
Mouse urine	3/29 (10.3)	4/24 (16.7)
Rat urine	3/28 (10.7)	2/23 (8.7)

* *p* = 0.004, Wilcoxon rank-sum test.

**Table 3:** Allergen levels from dust samples, by group and study visit.

		Mean ± SD (*n*)			Change from baseline^a^		
Allergen^b^	Visit	Control	Remediation	*p*-Value^c^	Control	Remediated	*p*-Value^d^
Cockroach (U/m^2^)	Baseline	−1.74 ± 2.13 (27)	−1.53 ± 1.72 (26)	0.40			
	EV2	−1.93 ± 1.60 (17)	−1.60 ± 1.64 (22)	0.59	0.38 ± 1.97	0.08 ± 1.73	0.79
	EV3	−1.44 ± 2.26 (17)	−1.40 ± 1.62 (16)	0.61	0.01 ± 1.78	−0.20 ± 2.22	0.67
Der f 1 (μg/m^2^)	Baseline	−1.89 ± 2.94 (27)	−2.36 ± 2.63 (26)	0.38			
	EV2	−2.37 ± 2.90 (17)	−2.55 ± 3.00 (22)	0.89	−0.79 ± 3.32	−0.56 ± 1.80	0.40
	EV3	−1.65 ± 2.98 (17)	−2.49 ± 3.47 (16)	0.61	0.53 ± 3.04	−0.85 ± 2.07	0.12
Der p 1 (μg/m^2^)	Baseline	−4.49 ± 2.52 (27)	−4.82 ± 2.14 (26)	0.65			
	EV2	−4.27 ± 2.52 (17)	−4.65 ± 2.31 (22)	0.50	0.14 ± 2.20*	0.11 ± 1.68	0.15
	EV3	−4.02 ± 1.78 (17)	−3.78 ± 2.93 (16)	0.86	0.53 ± 3.04	−0.85 ± 2.07	0.53
Der f 1 + Der p 1 (μg/m^2^)	Baseline	−1.25 ± 2.80 (27)	−2.08 ± 2.49 (26)	0.18			
	EV2	−1.91 ± 2.91 (17)	−2.27 ± 2.81 (22)	0.67	−0.91 ± 3.07	−0.53 ± 1.60	0.50
	EV3	−1.28 ± 2.65 (17)	−1.83 ± 3.22 (16)	0.91	0.17 ± 3.16	−0.48 ± 1.98	0.51
Endotoxin (μg/m^2^)	Baseline	0.36 ± 2.48 (28)	0.49 ± 1.69 (27)	0.70			
	EV2	0.23 ± 2.41 (17)	−0.46 ± 2.07 (24)	0.81	0.02 ± 3.67	−0.70 ± 1.80	0.03
	EV3	−0.59 ± 1.95 (18)	−0.62 ± 2.52 (19)	0.86	−0.41 ± 2.69	−0.76 ± 1.98	0.87
Mouse (ng/m^2^)	Baseline	2.35 ± 1.68 (27)	2.52 ± 0.98 (26)	0.66			
	EV2	2.39 ± 1.45 (17)	2.05 ± 1.69 (22)	0.48	0.24 ± 1.88	−0.43 ± 1.67	0.14
	EV3	2.11 ± 1.70 (17)	1.54 ± 1.99 (16)	0.35	−0.19 ± 1.54	−1.08 ± 1.99**	0.08
Rat (ng/m^2^)	Baseline	0.89 ± 1.63 (27)	1.38 ± 1.21 (26)	0.31			
	EV2	0.53 ± 1.54 (17)	0.56 ± 1.50 (22)	0.95	−0.90 ± 2.61	−0.83 ± 1.83	0.74
	EV3	0.15 ± 1.01 (17)	0.58 ± 1.45 (16)	0.28	−0.85 ± 1.84	−0.96 ± 2.06	0.91

a Within-group changes significantly different from zero (after adjusting for season of the year and surface type) are marked as follows:

* *p* < 0.05,

** *p* < 0. 01.

b Values reported are natural logarithms of values.

c Wilcoxon rank-sum test.

d Between-group test comparing mean changes, adjusting for season of the year, and surface type (carpet vs. hard surface).

**Table 4:** Mold scores [mean ± SD (*n*)].

Time point	Control	Remediation	*p*-Value^a^
Baseline (EV1)	3.03 ± 1.59 (33)	3.03 ± 2.16 (29)	0.66
6 months (EV2)	2.72 ± 1.99 (18)	1.38 ± 1.75 (26)	0.016
12 months (EV3)	1.68 ± 1.32 (22)	0.75 ± 0.99 (24)	0.009
Change EV2 − EV1	−0.28 ± 2.16 (18)	−1.42 ± 2.69 (26)	0.09
Test of change^b^	0.56	0.003	
Change EV3 − EV1	−1.45 ± 2.02 (22)	−2.58 ± 2.10 (24)	0.07
Test of change^b^	0.0003	< 0.0001	

a Between-group differences; Wilcoxon rank-sum test at EV1, EV2, EV3; test from mixed model adjusting for season of the year when comparing changes EV2 − EV1 and EV3 − EV1.

b Within-group differences; *p*-value for test of whether change is equal to zero, adjusting for season of the year.

**Table 5:** Comparison of mold indices between remediated and control groups.

		Mean ± SD^a^		Change from baseline estimate (95% CI)^b^	
Mold index^c^	Visit	Control	Remediated	Control	Remediated
Indoor molds	Baseline	6.45 ± 1.79	6.31 ± 1.39	—	—
	EV2	6.21 ± 1.43	5.59 ± 1.74	0.43 (−0.33 to 1.19)	−0.57 (−1.21 to 0.07)*
	EV3	6.35 ± 1.59	5.81 ± 1.80	0.33 (−0.40 to 1.04)	−0.41 (−1.08 to 0.27)
Outdoor molds	Baseline	8.79 ± 1.60	9.00 ± 1.17	—	—
	EV2	8.44 ± 1.50	8.31 ± 1.95	0.23 (−0.72 to 1.17)	−0.30 (−1.13 to 0.52)
	EV3	8.55 ± 1.83	8.39 ± 1.68	0.15 (−0.52 to 0.82)	−0.43 (−1.07 to 0.21)
Indoor:outdoor ratio	Baseline	−2.34 ± 1.18	−2.69 ± 0.89	—	—
	EV2	−2.23 ± 1.15	−2.72 ± 1.05	0.24 (−0.31 to 0.78)	−0.29 (−0.75 to 0.17)
	EV3	−2.20 ± 1.07	−2.59 ± 0.91	0.10 (−0.43 to 0.64)	−0.01 (−0.53 to 0.51)
Total fungal load	Baseline	7.19 ± 1.62	7.07 ± 1.27	—	—
	EV2	6.81 ± 1.39	6.40 ± 1.71	0.24 (−0.51 to 1.00)	−0.42 (−1.07 to 0.22)
	EV3	6.98 ± 1.55	6.54 ± 1.70	0.15 (−0.49 to 0.80)	−0.39 (−0.99 to 0.22)

a Sample sizes at baseline, EV2, and EV3 are 27, 18, and 18 for control and 26, 24, and 18 for remediated.

b Estimates are adjusted for season and surface type (carpet vs. hard surface) in a mixed linear regression model.

c Mold indices are defined as follows: Indoor molds = ln(geometric mean of indoor molds in cfu/m^2^). Outdoor molds = ln(geometric mean of outdoor molds in cfu/m^2^). Indoor:outdoor ratio = ln(geometric mean of indoor molds/geometric mean of outdoor molds) = ln(geometric mean of indoor molds in cfu/m^2^) − ln(geometric mean of outdoor molds in cfu/m^2^). Total fungal load = ln(geometric mean of indoor and outdoor molds, in cfu/m^2^). See “Materials and Methods” for complete list of indoor and outdoor molds.

* Differs from control group, *p* < 0.05.

**Table 6:** ED visits and hospitalizations during follow-up.

Time period	Outcome	Control (*n* = 33)	Remediation (*n* = 29)	*p*-Value
Entire 12 months of follow-up	≥ 1 ED or inpatient visits [*n* (%)]	12 (36.4)	5 (17.2)	0.15
	No. of ED/inpatient visits (mean ± SD)	0.91 ± 1.79	0.28 ± 0.80	0.06
From 6 months postrandomization to end of follow-up	≥ 1 ED or inpatient visits [*n* (%)]	11 (33.3)	1 (3.5)	0.003
	No. of ED/inpatient visits (mean ± SD)	0.52 ± 0.83	0.07 ± 0.37	0.004

*p*-Value from Fisher’s exact test or exact Wilcoxon rank-sum test.
